# Supplementary material for: Agrobacterium tumefaciens-Mediated Genetic Transformation of the Ect-endomycorrhizal Fungus Terfezia boudieri
Source: Genes (Basel). 2020 Oct 30;11(11):1293. doi: 10.3390/genes11111293 (PMC7693413; doi:10.3390/genes11111293)
Supplement: Supplementary file 1 [file genes-11-01293-s001.pdf]

Agrobacterium tumefaciens-mediated genetic transformation of the ect-endomycorrhizal fungus *Terfezia boudieri*

Lakkakula Satish [1,2], Madhu Kamle [1,4], Guy Keren [1], Galit Yehezkel [1], Ze'ev Barak [3], Varda Kagan-Zur [3], Ariel Kushmaro [2] and Yaron Sitrit [1*]

[1] The Albert Katz International School for Desert Studies, The Jacob Blaustein Institutes for Desert Research, Ben-Gurion University of the Negev, Beer Sheva - 84105, Israel. lsatish@post.bgu.ac.il (LS); guyguy123@gmail.com (GK); gality@bgu.ac.il (GY); sitrit@bgu.ac.il (YS)

[2] Avram and Stella Goldstein-Goren Department of Biotechnology Engineering and The Ilse Katz Center for Meso and Nanoscale Science and Technology, Ben-Gurion University of the Negev, Beer Sheva - 84105, Israel; Israel. lsatish@post.bgu.ac.il (LS); arielkus@bgu.ac.il (AK)

[3] Department of Life Sciences, Ben-Gurion University of the Negev, Beer Sheva - 84105, Israel. barakz@post.bgu.ac.il (ZB); zur@bgu.ac.il (VK)

[4] Department of Forestry, North Eastern Regional Institute of Science & Technology, Nirjuli, Arunachal Pradesh - 791109, India (current address). madhu.kamle18@gmail.com (MK)

* Correspondence: sitrit@bgu.ac.il

Supplementary figures

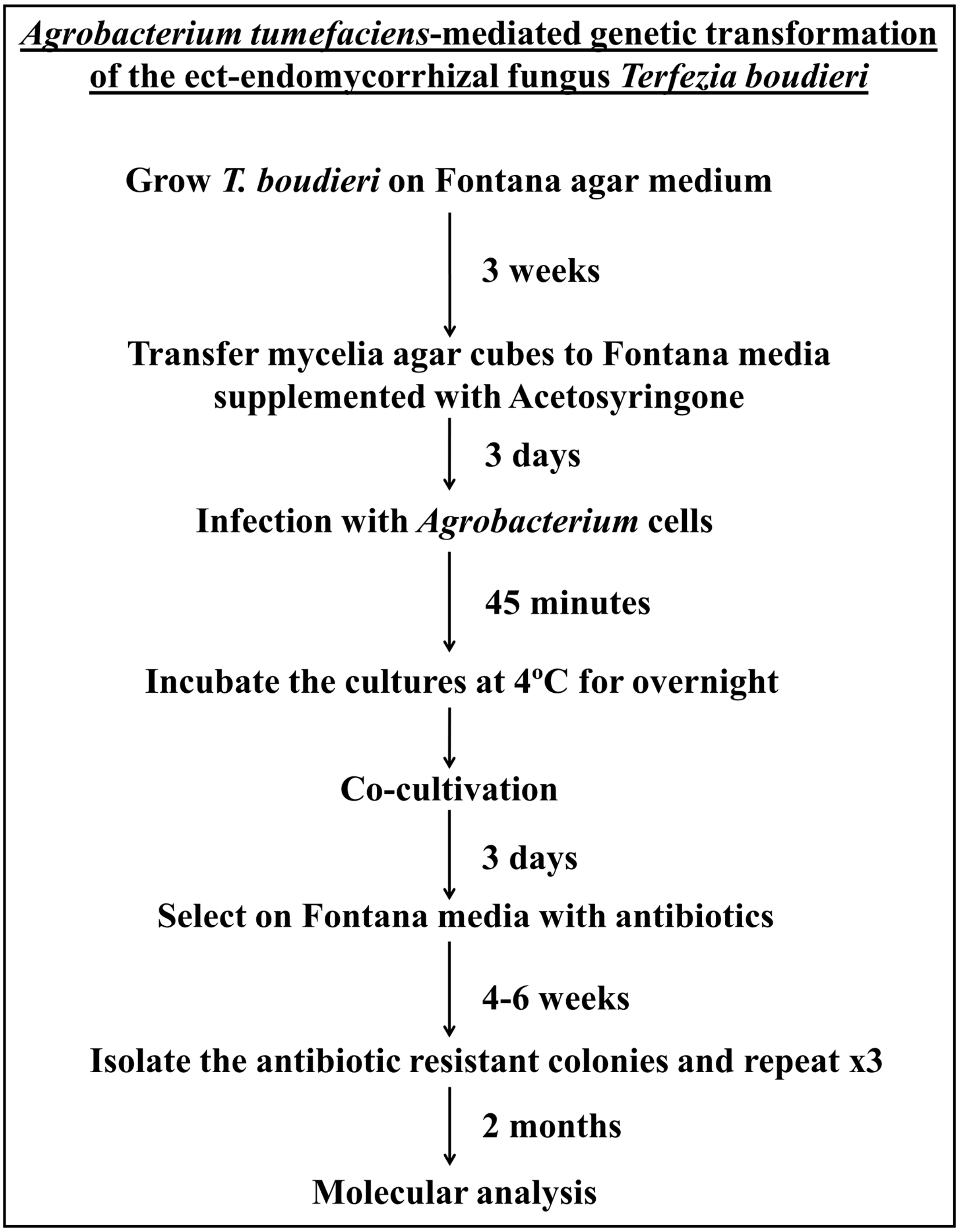

Figure S1. Flow chart of the transformation protocol for the desert truffle *T. boudieri*.

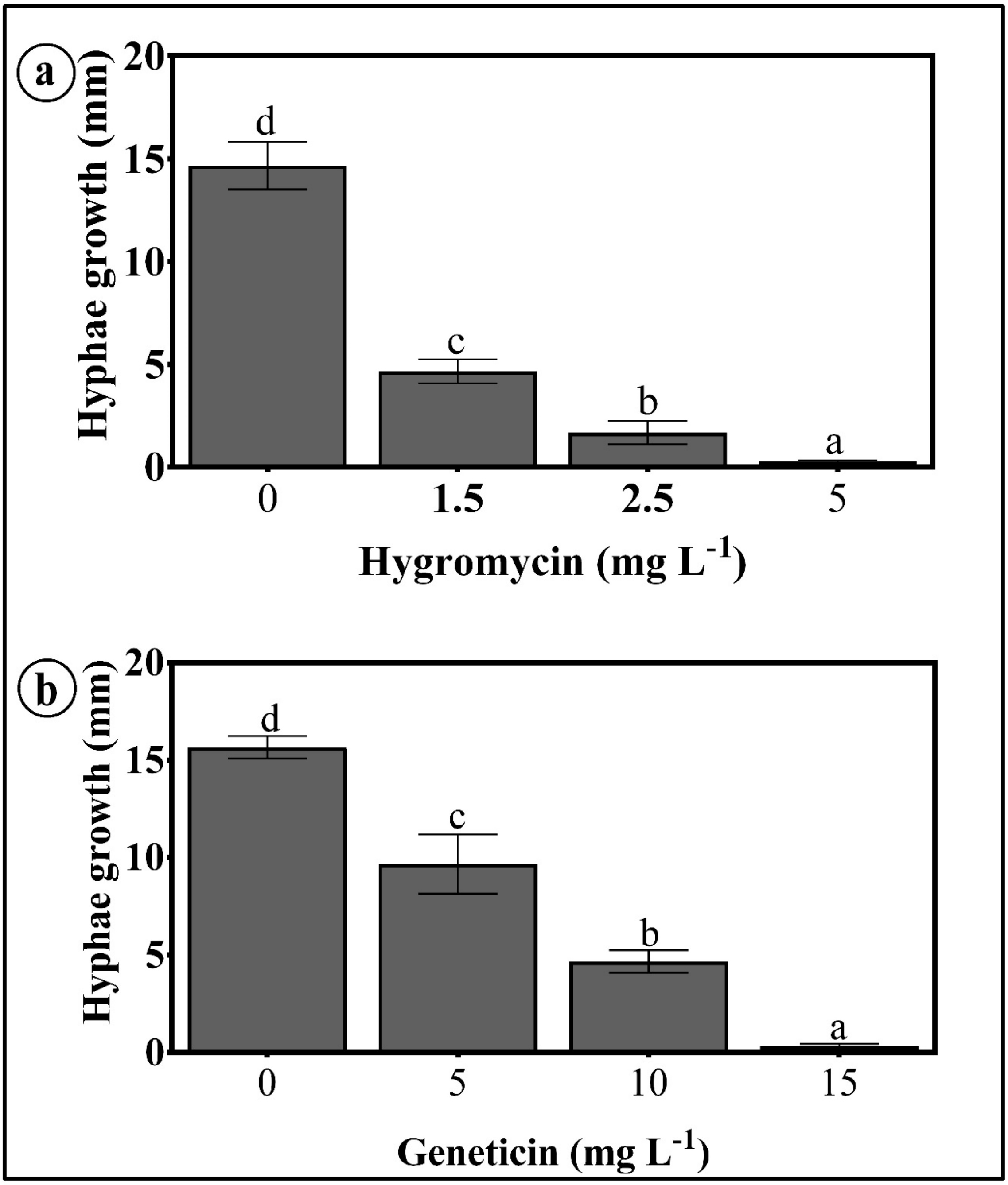

Figure S2. *T. boudieri* sensitivity to hygromycin and geneticin. Mycelial growth was determined after 2 weeks on Fontana medium supplemented with different concentrations of hygromycin (a) and geneticin (b). Values are mean ± SE (n=3), letters denote significant differences $p=0.05$ using Duncan's multiple range test.
